# Weight change among women using intramuscular depot medroxyprogesterone acetate, a copper intrauterine device, or a levonorgestrel implant for contraception: Findings from a randomised, multicentre, open-label trial

**DOI:** 10.1016/j.eclinm.2021.100800

**Published:** 2021-04-06

**Authors:** Mags Beksinska, Rodal Issema, Ivana Beesham, Tharnija Lalbahadur, Katherine Thomas, Charles Morrison, G.Justus Hofmeyr, Petrus S. Steyn, Nelly Mugo, Thesla Palanee-Phillips, Khatija Ahmed, Gonasagrie Nair, Jared M. Baeten, Jenni Smit

**Affiliations:** a MatCH Research Unit (MRU), Department of Obstetrics and Gynaecology, Faculty of Health Sciences, University of the Witwatersrand, Durban, South Africa; b Department of Epidemiology, University of Washington, Seattle, WA 98104, United States; c Department of Global Health, University of Washington, Seattle, WA 98104, United States; d FHI 360, Durham, NC, United States; e Effective Care Research Unit, University of the Witwatersrand, Walter Sisulu University, East London, South Africa; University of Botswana, Gaborone, Botswan; f UNDP-UNFPA-UNICEF-WHO-World Bank Special Programme of Research, Development and Research Training in Human Reproduction (HRP), World Health Organization, Geneva, Switzerland; g Center for Clinical Research (CCR), Kenya Medical Research Institute (KEMRI), Kenya; h University of the Witwatersrand, Wits Reproductive Health and HIV Institute (Wits RHI), Johannesburg, South Africa; i Setshaba Research Centre, Soshanguve, South Africa; j Faculty of Health Sciences, Department of Medical Microbiology, University of Pretoria; k Emavundleni Research Centre, Cape Town, South Africa

**Keywords:** Depot medroxyprogesterone acetate, Copper intrauterine device, Levonorgestrel implant, Contraception, Weight, Body mass index

## Abstract

**Background:**

There is limited evidence on the impact of the use of progestin-only hormonal contraception (POC) on weight change. We conducted a secondary analysis of prospective weight change among women enrolled in the Evidence for Contraceptive options and HIV Outcomes (ECHO) trial.

**Methods:**

The ECHO trial was conducted at 12 sites in eSwatini, Kenya, South Africa and Zambia between December 2015 and October 2018. HIV negative, women aged 16–35 years, desiring contraception, were randomised (1:1:1) to either 3-monthly intramuscular depot medroxyprogesterone acetate (DMPA-IM), levonorgestrel (LNG) implant or copper intrauterine device (IUD). Follow-up was up to 18 months. Weight (kg) was measured at baseline and study exit. Analysis was performed as intention to treat (ITT) and time on continuous contraceptive use. The primary outcome of this secondary analysis is weight change from study enrolment to the final visit at study month 12–18. The ECHO trial is registered with ClinicalTrials.gov, NCT02550067.

**Findings:**

7829 women were randomly assigned to DMPA-IM (*n* = 2609), copper IUD (*n* = 2607) or LNG implant (*n* = 2613). The ITT population included 7014 women 2293 DMPA-IM group, 2372 copper IUD group and 2349 LNG group) who were not lost to follow-up, pregnant on study, or missing weight data. The mean weight increased in all groups but was significantly different in magnitude: 3.5 kg (SD = 6.3), 2.4 kg (SD = 5.9) and 1.5 kg (SD = 5.7) in the DMPA-IM, LNG implant and copper IUD groups, respectively. Comparative differences between groups were (2.02 kg (95% CI, 1.68, 2.36, *p* < 0.001) for DMPA-IM versus copper IUD, 0.87 kg (0.53,1.20 *p* < 0.001) for LNG implant compared to copper IUD and 1.16 kg (0.82, 1.50, *p* < 0.001) for DMPA-IM compared with LNG implant. Results for continuous contraceptive use were similar.

**Interpretation:**

We found differences in weight gain between POC users compared to the non-hormonal copper IUD group over 12–18 months of use. Women using POCs should be counselled about this potential side effect when choosing a contraceptive method.

**Research in context panel, for all primary research Articles**

**Evidence before this study**

Progestin-only hormonal contraceptives (POC) use has been implicated in weight changes for many years, and is a frequent reason for method discontinuation. Although many studies report weight gain in users of a range of POCs, the most recent *Cochrane* systematic review published in 2016 found that there was insufficient evidence to determine the effect of POCs on weight. Studies investigating weight changes in POC users have been limited due to the lack of a non-hormonal comparison group, lack of randomisation and poor continuation rates.

**Added value of this study**

This is the largest randomised trial to date where two POC methods have been compared to a non-hormonal method. High retention and randomised method continuation allows our data to show that there are real differences in weight gain between POC users compared to non-hormonal method users over a 12–18 month period of use.

**Implications of all the available evidence**

The results of this analysis will have important implications for contraceptive programme management as injectables and implants are widely used globally and are the most commonly used methods in Sub-Saharan Africa making up over half of all modern contraceptive use. Not all women using POCs in our study gained weight, and this should be made clear in contraceptive counselling messages. Women seeking effective contraception, such as POCs should not be deterred from using these methods, and should be presented with the available data.

Alt-text: Unlabelled box

## 1 Introduction

Progestin-only hormonal contraception (POC) is available in several forms including injectables, implants, levonorgestrel-releasing intrauterine contraception (LNG-IUD), and oral contraceptives. Injectables and implants are widely used globally and are the most commonly used methods in Sub-Saharan Africa, making up over half of all modern contraceptive use [1]. POC use has been implicated in weight changes and weight gain is commonly cited as a side effect by users and providers [2], [3], [4], and is a frequent reason cited for method discontinuation [4], [5], [6], [7]. This concern can additionally deter women from initiation of POCs [7], despite their safety and reliability.

Although many studies report weight gain in users of a range of POCs [[2], [3], [4],^6^,[8], [9], [10], [11], [12], [13], [14], [15], [16], [17], [18], [19], [20]], the literature has focused primarily on depot medroxyprogesterone acetate (DMPA) delivered as an intramuscular injection 150 mg/ml (IM) with studies reporting weight gain of up to 2–3 kg in the first year of use [[8], [9], [10],^16^,^20^,^21^], followed by gains of between 4 and 10 kg with longer term use of 3–5 years [2,15,[17], [18], [19]].

In a non-randomised trial, the second generation two rod LNG implant Jadelle® was found to have induced similar weight increases to the single rod etonogestrel (ENG) implant Implanon®, with weight gains of approximately 3 vs 1 kg for the Copper IUD users over a 3 year period [22]. In another study, two rod LNG implant users showed greater weight gain compared to the ENG implant users [13]. Other studies have found no differences between the ENG implant compared to other methods [23,24]. Similarly, limited data are available for intrauterine and oral POCs which show small or insignificant changes in weight, although in some studies an increase in fat mass was found in users of both these methods compared to non-hormonal user controls [11,16,22,24].

The most recent *Cochrane* systematic review published in 2016 found that there was insufficient evidence from randomised trials to determine the effect of POCs on weight [7]. The review assessed studies reporting change in body weight or other body measure of lean or fat mass in POCs users compared with another contraceptive method or no contraceptive. The review concluded that there was little evidence of weight gain when using POCs, with a mean weight gain at 6 or 12 months of less than 2 kg in most studies. Non-POC comparison groups were found to have similar weight gains.

Studies investigating weight changes in POC users have been limited for several reasons:- the lack of a non-hormonal comparison group, lack of randomisation and poor continuation rates. Weight gain or loss (perceived or real) has resulted in women discontinuing from trials and potentially biasing results [17,22]. To date, all these factors have limited the availability of high quality evidence enabling few conclusions to be drawn on the effect of POC use on weight change.

The Evidence for Contraceptive options and HIV Outcomes (ECHO) trial was an open label, prospective randomised multicentre trial which compared the risk of HIV acquisition among women randomised to DMPA-IM, the levonorgestrel (LNG) implant or the copper IUD [25]. We conducted a secondary analysis of weight data collected, to describe and compare changes in weight and body mass index (BMI) between women randomised to these three contraceptive methods, two of which were POCs.

## 2 Methods

### 2.1 Study design and participants

This randomised multi-centre trial was conducted in 12 research sites in four African countries. Nine sites in South Africa, and one site each in Kenya, eSwatini and Zambia participated in the trial which was conducted between December 2015 and October 2018. Women were invited to enrol into the ECHO trial if they desired effective family planning and were willing to be randomised to any one of the three trial contraceptive methods (DMPA-IM, the LNG implant or the copper IUD). Women were eligible if they were not pregnant, were HIV-seronegative, aged 16–35 years, had no medical contraindications to the trial contraceptive methods, were willing to use their assigned method for 18 months, reported not using injectable, intrauterine, or implantable contraception for the previous six months and reported being sexually active. Follow-up visits occurred at 1 month, 3 months and quarterly (every 3 months) thereafter up to 12, 15 and 18 months.

At baseline (inclusive of the screening and enrolment visits), demographic, sexual and reproductive risk behaviour, and reproductive and contraceptive history were collected. Weight and height were measured at baseline and exit visits according to a standardised protocol, using calibrated equipment across all sites. Weight was measured to 0.1 kg and height measured to the nearest cm. Body mass index (BMI) was calculated (kg/m^2^). Every follow-up visit included assessment of randomised contraceptive method use, HIV serological testing, safety monitoring and behavioural assessments. Participants received a comprehensive package of HIV prevention services and contraceptive counselling. The study design and primary results have been previously reported [25].

### 2.2 Randomisation, masking and procedures

At enrolment, women were randomly assigned (1:1:1) to the DMPA-IM group, Copper IUD group, or LNG implant group. Participants received a Cu IUD (Optima TCu380A; Injeflex, Sao Paolo, Brazil), an LNG implant (Jadelle; Bayer, Turku, Finland) or an injection of 150 mg/mL DMPA-IM (Depo Provera; Pfizer, Puurs, Belgium), which was provided on site at enrolment and for the DMPA-IM group at every 3-monthly follow-up visit up to 18 months.

Ethics review committees at each study site, FHI 360, and the World Health Organization (WHO) approved the study protocol. Women provided written informed consent in a language of their choice prior to the conduct of any study related procedures.

Outcomes: The primary outcome of this secondary analysis is weight change from study enrolment to the final visit at study month 12–18.

### 2.3 Statistical analysis

To examine our hypothesis that POC use may increase weight over time, we used linear regression to examine the effect of randomised contraceptive method on weight at final visit, adjusting for weight at baseline, site, and study month of final visit (to account for exit visits varying from 12/15/18 months). The analysis was performed in two ways: (1) as an intention to treat (ITT) analysis, in which final visit data on all women was included, even if they discontinued their randomised method prior to the final visit; (2) as a “Continuous Use” analysis including only women who continued their randomised method through their final visit. Women were considered to have discontinued their randomised method as follows; for those randomised to DMPA, if they did not start on the day of enrolment or more than 119 days elapsed between injections; for those randomised to implant, if not started on the day of enrolment or  ≥ 1 day elapsed before reinsertion after removal for any reason; or for Copper IUD if more than 30 days elapsed before initial insertion, or more than 28 days elapsed before re-insertion after an expulsion, or  ≥ 1 day elapsed before re-insertion following removal for any reason. In both ITT and continuous use analyses, women were excluded from the analysis if pregnant during the study, up to and including at the final visit.

To explore whether the effect of randomised contraceptive use varied by baseline factors of BMI category (underweight < 18.5, normal 18.5–24.9, pre-obesity 25–29.9, obesity 30–34.99, severe obesity 35–39.9, morbid obesity > 40); age category (< 25 years, >/= 25 years); or report of prior history of DMPA use category, we added to the model main effect of BMI (or age/prior DMPA-IM history) and the interaction term between BMI (age/DMPA-IM history) and randomisation group. If the interaction term was statistically significant, we interpreted this to mean the effect of group was modified by participants’ baseline BMI (age/DMPA-IM history). *P*-values and confidence intervals were not adjusted for multiple testing.

To explore patterns of weight changes over time in each group, we described weight changes by group in those exiting at 12, 15, and 18 month visits. To explore whether there was a constant rate of weight change over time, for each group we modelled weight gain per month on study at exit. We added a squared term for month at exit to the model to test for evidence of a nonlinear (nonconstant) change in weight over time. Analyses were done with SAS version 9.4 and R, version 3.4.1.

This study is registered with ClinicalTrials.gov, number NCT02550067.

### 2.4 Role of the funding source

The study funders and manufacturers played no role in the design, collection, analysis and interpretation of the data and in the decision to submit this paper for publication.

The authors were involved in the data collection, performed all analyses, vouch for the data completeness, prepared the manuscript, and were responsible for the decision to submit for publication. Katherine Thomas, Rodal Issema and Jared Beaton had full access to all the data in the study. Individual site investigators had access to the dataset from their own site and to analysed data from other sites.

## 3 Results

A total of 7830 women were enrolled across the 12 trial sites. Of these women, 7829 were randomly assigned to the following contraceptive methods:- DMPA-IM 2609, copper IUD 2607 and LNG implant 2613. Follow-up was up to 18 months with the later enrolling participants contributing 12–15 months of follow-up. Almost all (99%) women accepted their randomised method at enrolment and more than 91% of women attended each scheduled visit to the end of follow-up in each study group.

**Fig. 1:**
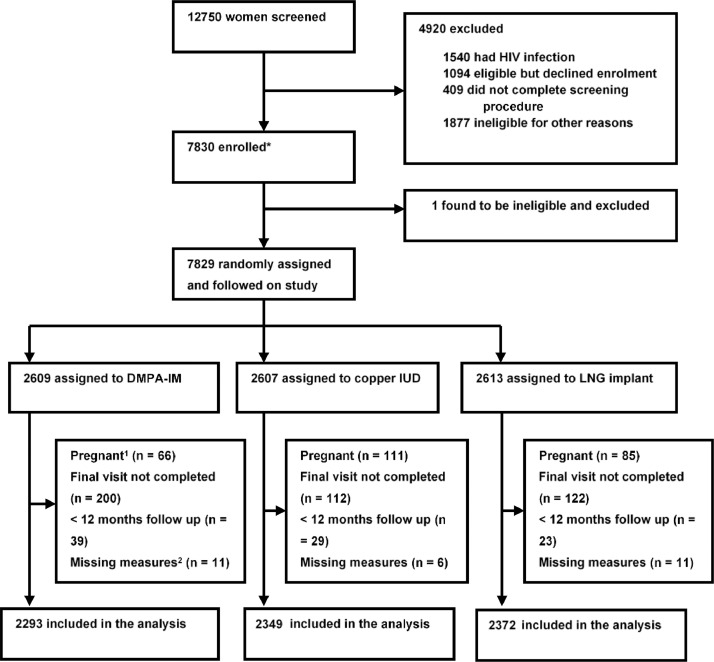
Trial profile. 1. Woman who were pregnant at any time during the study. 2. Missed a baseline or exit visit weight/height or data entry error that could not be resolved.

The final analysis sample size included 7014/7829 (89.6%) of randomised women (Fig. 1). A total of 815 women (10.4%) were excluded from analysis for the following reasons: did not complete their scheduled exit visit (*n* = 434, 5.5%); exited or weight taken for another reason but before 12 month visit (*n* = 91); were pregnant at any time during the study, including a small number estimated to have been already been pregnant at study enrolment (*n* = 262); or missed a baseline weight, baseline/exit height measurement or had a possible data entry error that could not be resolved (*n* = 28). The final ITT population consisted of 2293 assigned to DMPA-IM, 2349 to copper IUD and 2372 to LNG implant. In total 70.2% had completed 18 months follow -up, 19.8% 15 months and 10.0% 12 months.

Baseline demographic characteristics of the final analysed population are shown in Table 1. Just under two-thirds (61.8%) of women were aged 18–25 years. Only one study site (Kenya) enrolled women between 16–18 years, resulting in a small sample of women, 57 (0.8%) under 18 years. The proportion of women in each BMI category at enrollment was similar across the randomised groups. Just over half, (*n* = 3680, 52.4%) had a BMI at baseline that classified them in one of the obesity categories (including pre-obesity) with 303 women (4.0%) having morbid obesity. Less than 5% in each group were underweight. Characteristics of women and their distribution across the groups were similar to the total enrolled study population [25].

**Table 1:** Baseline characteristics of analysed participants by randomised group (intramuscular depot medroxyprogesterone acetate, levonorgestrel implant or copper intrauterine device), ITT population N (%).

	DMPA-IM (*n* = 2293)	LNG Implant (*n* = 2372)	Copper IUD (*n* = 2349)	Total(*n* = 7014)
**Age (years)**				
16–17	13 (0.6%)	20 (0.8%)	24 (1.0%)	57 (0.8%)
18–20	599 (26.1%)	623 (26.3%)	609 (25.9%)	1831 (26.1%)
21–24	837 (36.5%)	848 (35.8%)	816 (34.7%)	2501 (35.7%)
25–30	642 (28.0%)	676 (28.5%)	686 (29.2%)	2004 (28.6%)
31+	202 (8.8%)	205 (8.6%)	214 (9.1%)	621 (8.9%)
**Marital status**				
Never Married	1819 (79.3%)	1886 (79.5%)	1878 (79.9%)	5583 (79.6%)
Married	456 (19.9%)	464 (19.6%)	459 (19.5%)	1379 (19.7%)
Previously Married	18 (0.8%)	22 (0.9%)	12 (0.5%)	52 (0.7%)
**Lives with partner**	685 (30.1%)	700 (29.8%)	709 (30.4%)	2094 (30.1%)
**Education**				
No schooling	15 (0.7%)	18 (0.8%)	11 (0.5%)	44 (0.6%)
Primary school	192 (8.4%)	240 (10.1%)	222 (9.5%)	654 (9.3%)
Secondary school	1742 (76.0%)	1744 (73.5%)	1743 (74.2%)	5229 (74.6%)
Post-secondary school	344 (15.0%)	370 (15.6%)	373 (15.9%)	1087 (15.5%)
**Any prior pregnancy**	1877 (81.9%)	1970 (83.1%)	1925 (81.9%)	5772 (82.3%)
**BMI (kg/m²)**				
Underweight (<18.5)	109 (4.8%)	91 (3.8%)	103 (4.4%)	303 (4.3%)
Normal (18.5–24.9)	1018 (44.4%)	991 (41.8%)	1021 (43.5%)	3030 (43.2%)
Pre-obesity (25–29.9)	581 (25.3%)	627 (26.4%)	616 (26.2%)	1824 (26.0%)
Obesity (30–34.9)	325 (14.2%)	401 (16.9%)	334 (14.2%)	1060 (15.1%)
Severe obesity (35–39.9)	169 (7.4%)	171 (7.2%)	172 (7.3%)	512 (7.3%)
Morbid obesity (40+)	91 (4.0%)	90 (3.8%)	103 (4.4%)	284 (4.0%)

The mean weight difference from baseline to final visit indicated an overall increase in weight across all three groups with the largest gain in the DMPA-IM group of 3.5 kg, 2.4 kg in the LNG implant and 1.5 kg in the copper IUD group (Table 2). Similarly, BMI increase at final visit was highest in the DMPA-IM group. In terms of percent increase in weight, we observed a 5.5%, 3.7% and 2.5% gain for DMPA-IM, LNG implant, and copper IUD, respectively.

**Table 2:** Weight and BMI changes by group (intramuscular depot medroxyprogesterone acetate, levonorgestrel implant or copper intrauterine device), ITT population, Mean or N(%).

	DMPA-IM *n* = 2293	LNG Implant *n* = 2372	Copper IUD *n* = 2349	Total *n* = 7014
**Weight**				
Baseline weight	67.5 (16.9)	68.4 (16.5)	67.7 (17.3)	67.9 (16.9)
Final weight	71.0 (18.1)	70.8 (17.5)	69.2 (18.0)	70.3 (17.9)
Weight difference^1^	3.5 (6.3)	2.4 (5.9)	1.5 (5.7)	2.5 (6.0)
% Weight change^2^	5.5 (9.3)	3.7 (8.7)	2.5 (8.6)	3.8 (9.0)
***Weight difference categories***				
Lost 5+ kg	147 (6.4%)	211 (8.9%)	242 (10.3%)	600 (8.6%)
Lost 2 to 4.9 kg	233 (10.2%)	297 (12.5%)	365 (15.5%)	895 (12.8%)
Gained/lost less than 2 kg	518 (22.6%)	642 (27.1%)	662 (28.2%)	1822 (26.0%)
Gained 2 to 4.9 kg	517 (22.5%)	521 (22.0%)	483 (20.6%)	1521 (21.7%)
Gained 5 to 9.9 kg	572 (24.9%)	472 (19.9%)	446 (19.0%)	1490 (21.2%)
Gained 10+ kg	306 (13.3%)	229 (9.7%)	151 (6.4%)	686 (9.8%)
**BMI(kg/m^2^)**				
Baseline BMI	26.5 (6.4)	26.9 (6.4)	26.7 (6.6)	26.7 (6.4)
Final BMI	27.9 (6.9)	27.8 (6.8)	27.3 (6.9)	27.7 (6.9)
BMI difference^1^	1.4 (2.4)	0.9 (2.3)	0.6 (2.3)	1.0 (2.4)
% BMI change^2^	5.5 (9.3)	3.7 (8.7)	2.5 (8.6)	3.8 (9.0)
***Baseline BMI categories***				
Underweight (< 18.5)	110 (4.8%)	95 (4.0%)	105 (4.5%)	310 (4.4%)
Normal (18.5–24.9)	1030 (44.9%)	996 (42.0%)	1039 (44.2%)	3065 (43.7%)
Pre-obesity (25–29.9)	577 (25.2%)	628 (26.5%)	602 (25.6%)	1807 (25.8%)
Obesity (30–34.9)	318 (13.9%)	396 (16.7%)	328 (14.0%)	1042 (14.9%)
Severe obesity (35–39.9)	168 (7.3%)	170 (7.2%)	175 (7.4%)	513 (7.3%)
Morbid obesity (40+)	90 (3.9%)	87 (3.7%)	100 (4.3%)	277 (3.9%)
***Final BMI categories***				
Underweight (<18.5)	93 (4.1%)	91 (3.8%)	106 (4.5%)	290 (4.1%)
Normal (18.5–24.9)	836 (36.5%)	884 (37.3%)	939 (40.0%)	2659 (37.9%)
Pre-obesity (25–29.9)	600 (26.2%)	618 (26.1%)	625 (26.6%)	1843 (26.3%)
Obesity (30–34.9)	411 (17.9%)	429 (18.1%)	364 (15.5%)	1204 (17.2%)
Severe obesity (35–39.9)	204 (8.9%)	224 (9.4%)	179 (7.6%)	607 (8.7%)
Morbid obesity (40+)	149 (6.5%)	126 (5.3%)	136 (5.8%)	411 (5.9%)

*Statistics presented: mean (SD); *n* (%).

1 Last minus First measure.

2 (Last measure - First measure)/First measure.

About a quarter (26.0%) of women overall gained or lost less than 2 kg over the follow-up period. A third (38.2%) of women in the DMPA-IM group gained at least 5 kg over the follow-up period compared to 29.6% of the LNG-implant group and 25.4% of the copper IUD group. A quarter (25.8%) of women in the copper IUD group lost weight (2 kg or more) with a lower proportion of women losing weight in the LNG implant group (21.4%), while fewest (16.6%) women in the DMPA-IM group lost weight.

The observed weight change was significantly different between the three groups in both the ITT and continuous use analyses (Table 3). ITT analysis showed a mean kg difference (95% CI) of 2.02 (1.68, 2.36; *p* < 0.001), for DMPA-IM compared with copper IUD, 0.87 (0.53, 1.20; *p* < 0.001), for LNG implant compared to copper IUD, and 1.16 (0.82, 1.50); *p* < 0.001), for DMPA-IM compared with LNG implant. Continuous use results were very similar, with mean kg difference of 2.30 (1.92, 2.67) for DMPA-IM compared with copper IUD, 1.05 (0.70, 1.41) for LNG implant compared to copper IUD, and 1.24 (0.87, 1.62) for DMPA-IM compared with LNG implant, all comparisons *p* < 0.001. Age modified the effect of DMPA-IM compared with LNG implant, with lower gains in weight seen in women aged ≥ 25 in the LNG implant group, compared to those <25 years, while in the DMPA-IM group, weight increase was higher and similar in both age groups (additional increase on DMPA-IM compared to LNG implant  = 1.68 kg in women ≥ 25 years vs. 0.86 kg in women < 25 years, *p* = 0.022 for interaction). There was no significant interaction by age between DMPA-IM vs. the copper IUD, or the LNG implant vs. the copper IUD. Baseline BMI categorised as either underweight, normal or pre-obesity (< 30 kg/m²) compared to those in the obesity categories (≥ 30 kg/m²) had no significant impact on the effects of the contraceptive methods on weight change. Similarly, prior DMPA use at baseline showed no effect on the effects of contraceptive methods on weight change.

**Table 3:** Mean weight changes and statistical comparisons by group (intramuscular depot medroxyprogesterone acetate, levonorgestrel implant or copper intrauterine device).

	DMPA-IM		LNG Implant		Copper IUD		DMPA-IM vs Copper IUD		LNG Implant vs Copper IUD		DMPA-IM vs LNG Implant	
	N	Mean Weight Gain, (kg) (95% CI)	N	Mean Weight Gain, (kg) (95% CI)	N	Mean Weight Gain, (kg) (95% CI)	Mean difference, kg (95% CI)	*p* value	Mean difference, kg (95% CI)	*p* value	Mean difference, kg (95% CI)	*p* value
**ITT**	2293	3.50 (3.24, 3.76)	2372	2.37 (2.13, 2.61)	2349	1.51 (1.28, 1.74)	2.02 (1.68, 2.36)	< 0.001	0.87 (0.53, 1.20)	< 0.001	1.16 (0.82, 1.50)	< 0.001
**Continuous use**	1666	3.61 (3.32, 3.90)	2132	2.45 (2.19, 2.70)	2037	1.40 (1.15, 1.65)	2.30 (1.92, 2.67)	< 0.001	1.05 (0.70, 1.41)	< 0.001	1.24 (0.87, 1.62)	< 0.001
**Age, years**								0.176*		0.344*		0.022*
< 25	1451	3.46 (3.13, 3.78)	1492	2.65 (2.36, 2.94)	1450	1.67 (1.38, 1.95)	1.84 (1.41, 2.27)		0.98 (0.56, 1.41)		0.86 (0.43, 1.28)	
25+	842	3.58 (3.17, 4.00)	880	1.89 (1.48, 2.30)	899	1.26 (0.87, 1.65)	2.32 (1.77, 2.88)		0.65 (0.10, 1.20)		1.68 (1.12, 2.24)	
**Baseline BMI (kg/m²)**								0.162*		0.609*		0.363*
<Obesity (<30)	1709	3.46 (3.19, 3.74)	1711	2.38 (2.12, 2.65)	1740	1.58 (1.33, 1.83)	1.88 (1.49, 2.28)		0.81 (0.42, 1.21)		1.07 (0.67, 1.46)	
Obesity+ (30+)	584	3.62 (3.00, 4.24)	661	2.33 (1.80, 2.85)	609	1.31 (0.79, 1.84)	2.44 (1.76, 3.11)		1.01 (0.36, 1.67)		1.42 (0.76, 2.08)	
**Prior DMPA use**								0.329*		0.563*		0.120*
No	1144	3.23 (2.87, 3.59)	1151	2.35 (2.01, 2.69)	1132	1.39 (1.06, 1.71)	1.86 (1.37, 2.34)		0.97 (0.48, 1.46)		0.89 (0.40, 1.37)	
Yes	1149	3.77 (3.40, 4.14)	1221	2.38 (2.05, 2.72)	1217	1.63 (1.30, 1.96)	2.20 (1.72, 2.67)		0.77 (0.30, 1.24)		1.43 (0.95, 1.90)	

Controlled for woman's weight at baseline, study visit, and study site.

⁎ *p* value is for interaction term which indicates whether mean difference between arms, varies by subgroup. Subgroup-specific mean differences are provided with 95% CIs. Subgroup analyses are ITT.

We assessed the differences in weight in women who exited the study at 12, 15 and 18 months (Table 4). Women in the DMPA-IM group who exited at 12 months had gained an average of 2.8 kg, at 15 months, 3.7 kg, and at 18 months, 3.6 kg; while weight gain was approximately 0.21 kg per month over the 18 month period, there was modest evidence that the rate of gain was not consistent but levelled off between 12 and 18 months (*p* = 0.74 for nonlinear pattern of weight gain). Weight gain in those exiting at 12 months in the LNG implant group was 1.2 kg, at 15 months 1.9, and at 18 months, 2.7 kg. In LNG implant users exiting at 18 months, an overall rate of weight gain of approximately 0.14 kg/month was observed. Women exiting at 12 months in the LNG implant and the copper IUD had gained a similar amount of weight, but in the copper IUD group those exiting at 15 and 18 months gained little or no additional weight; consistent with this observation we found that weight gain over time did not appear to be linear in the copper IUD group (*p* = 0.03 for nonlinear pattern of weight gain).

**Table 4:** Mean Weight changes by group (intramuscular depot medroxyprogesterone acetate, levonorgestrel implant or copper intrauterine device) by month of final weight measurement.

	DMPA-IM *n* = 2293	LNG Implant *n* = 2372	Copper IUD *n* = 2349	Total *n* = 7014
*12 months*	*n = 229*	*n = 236*	*n = 239*	*n = 704*
**Baseline weight**	67.9 (17.2)	67.8 (16.2)	69.5 (17.7)	68.4 (17.0)
**Final weight**	70.2 (17.7)	69.0 (17.1)	70.9 (19.0)	70.0 (18.0)
**Weight difference**^1^	2.2 (5.4)	1.2 (5.3)	1.5 (4.9)	1.6 (5.2)
**% Weight change**^2^	3.5 (7.9)	1.8 (7.7)	2.0 (7.0)	2.4 (7.6)
*15 months*	*n = 449*	*n = 466*	*n = 471*	*n = 1386*
**Baseline weight**	66.4 (16.1)	68.4 (17.0)	67.3 (17.2)	67.4 (16.8)
**Final weight**	70.1 (17.3)	70.3 (18.0)	68.6 (17.9)	69.7 (17.7)
**Weight difference**^1^	3.7 (5.8)	1.9 (5.5)	1.3 (5.9)	2.3 (5.8)
**% Weight change**^2^	5.8 (9.4)	2.9 (8.1)	2.2 (8.9)	3.6 (8.9)
*18 months*	*n = 1615*	*n = 1670*	*n = 1639*	*n = 4924*
**Baseline weight**	67.7 (17.1)	68.5 (16.3)	67.6 (17.3)	67.9 (16.9)
**Final weight**	71.4 (18.4)	71.1 (17.4)	69.1 (17.9)	70.6 (17.9)
**Weight difference**^1^	3.6 (6.5)	2.7 (6.1)	1.6 (5.8)	2.6 (6.2)
**% Weight change**^2^	5.7 (9.5)	4.1 (9.0)	2.6 (8.7)	4.1 (9.2)
**Nonlinear change over time within group**^3^	*p* = 0.74	*p* = 0.65	*p* = 0.03	n/a

*Statistics presented: mean (SD).

1 Last minus First measure.

2 (Last measure - First measure)/First measure.

3 Models with linear term for month were assessed for whether adding a squared term for month was statistically significant. Statistically significant squared term for month was interpreted to mean the relationship was nonlinear, i.e., the rate of weight change over time was not constant but rather increased or decreased over time.

**Fig. 2:**
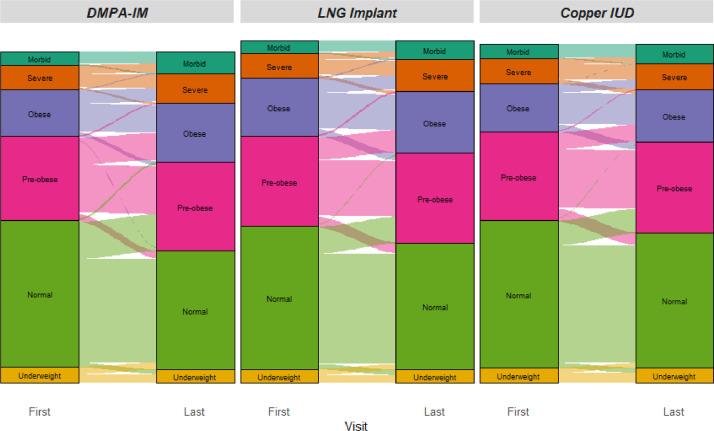
Shifts in BMI categories from baseline to final measurement. Fig. 2 displays the number of women within each randomized arm falling into each BMI category at baseline and at final measurement; the proportion of women transitioning from one category to another over time are shown as lighter-intensity paths between baseline and final visit, with the thickness of each path indicating the size of the group falling into a given transition between categories from baseline to final visit.

Changes in BMI categories among women from their baseline to final visit are shown for each randomised contraceptive group in Fig. 2 with additional data shown in supplementary Table 2. Women in all three groups show sizeable proportions who changed categories. Consistent with the highest overall change being seen in women randomised to DMPA-IM, women in that group who changed from categories over time mostly changed to a higher category as shown by thicker connecting lines moving upwards. LNG implant also shows this pattern but to a lesser extent, and Copper IUD showed women more equally moving up or down from their starting category.

Self- reported weight increase was cited as a reason for method discontinuation by a total of 19 women using DMPA-IM and seven LNG-Implant users, while no copper IUD users discontinued for this reason. Conversely, 18 women in the LNG-implant group discontinued for self-reported abnormal weight loss while only 3 women in each of the DMPA-IM and copper-IUD groups gave this reason.

## 4 Discussion

The strength of our study is that it is a secondary analysis nested within the largest randomised trial to date where POCs have been compared to a non-hormonal contraceptive method. The ECHO trial's high retention and randomised method continuation allows our data to show true differences in both ITT and continuous use analyses in weight gain between POC users compared to non-hormonal method users as well as differences between the two POC methods over a 12–18 month period of use.

Our study found an average increase in weight across all three methods during the study follow-up. Weight gain was highest in the DMPA-IM group followed by the LNG implant, while the Copper IUD users gained least weight over time. It is important to note that not all women gained weight and a small proportion lost weight, however depending on method, almost a quarter to over a third of all women gained at least 5 kg during follow-up. The average weight gain in DMPA-IM users of 3.5 kg is comparable with other studies that have presented average weight differences over similar lengths of follow-up [[8], [9], [10],^16^,^20^,^21^]. The average weight gain of 2.4 kg in the two rod LNG implant group is slightly higher than that reported in previous studies [22,26].

It is important to identify characteristics of women who are at risk of weight gain, and some studies have suggested that baseline weight may influence prospective weight in women using POCs [8,20], although other studies have not found this to be the case [12,15,17,19].

Our study found no effect of baseline weight on endline weight gain. Another risk factor previously identified for DMPA-IM is that those women experiencing early weight gain may be at risk of greater weight gain, with continued use [2,27]. We were unable to assess this in our study as weight was only collected at enrolment and at the study exit visit. This lack of consensus from previous studies may be in part due to poor study continuation rates. Follow-up of adolescents and young women in contraceptive studies can be challenging as contraceptive discontinuation rates are high. In one study of adolescents, over one-third (37%) of DMPA-IM users had discontinued the study by 18 months [8]. In another study that included a mix of adolescents and young women with a mean age of 24 years using COCs or DMPA-IM, 73% of women did not complete the 3-year study for a number of reasons [19]. Although many were lost to follow-up, 19% wanted a different method of contraception. This indicates the importance of following-up women beyond discontinuation, not only to collect reasons for method discontinuation but also to monitor weight changes. This would give us a better understanding of weight change post discontinuation of POCs.

Although our study was limited to a baseline and a single exit weight measurement at either 12, 15 or 18 months, our data do appear to show that women in the DMPA-IM and LNG implant groups continue to gain weight after the first 12 months. Although there was a significant change in weight gain at 12 months in the Copper IUD users, no significant further weight gain occurred at 15 and 18 months.

The role of progestins and estrogens in hormonal contraception, and the possible mechanisms through which weight change could occur, are complex [14]. Most research on the mechanism of weight gain in contraceptive users has been conducted with DMPA-IM users. A one-year follow-up study found increases in Leptin (a hormone that controls appetite which is found in higher levels in women with obesity) in DMPA users compared to copper IUD users in those gaining > 3 kg [28].

Data from South Africa has suggested weight gain in women associated with use of antiretroviral regimens [29]. As POCs are the most commonly used contraceptive methods in South Africa and nine sites in the ECHO study were from this country, the combined effect of POCs and use of antiretrovirals should be further investigated.

Reporting of mean weight change alone may not be as useful as more detailed information about proportions of women who decrease, increase or remain stable in terms of weight and BMI. It is also important for studies to report the percent change of weight from baseline, and, in addition, present more detail on subgroups of different baseline weights and the proportion of women gaining and losing weight.

Finally, we should be cognisant that not all women using POCs in our study gained weight, and this should be made clear in contraceptive guidance messages. Women seeking effective contraception, such as POCs should not be deterred from using these methods, and should be presented with the available data so that they can make an informed decision about which contraceptive methods to use. Additional research should be undertaken to understand the mechanism of weight changes associated with the two POCs used in this study.

Our study only included two POC methods and there exists a lack of consistent evidence on other methods. For example, there is minimal information on weight changes in a range of POCs such as DMPA-SC, NET-EN (2- month injection) and oral POCs, although some of these methods have been available for many years. Weight was collected at enrolment and exit visits in this study and this limits our ability to examine detailed changes in weight over shorter time periods. However, two sites in South Africa collected weight at each 3 monthly follow-up and these data will be analysed to understand more nuanced patterns of weight change over time.

## Declaration of Competing Interest

Dr. Baeten reports grants from USAID, grants from BMGF, during the conduct of the study; personal fees from Gilead Sciences, outside the submitted work; Dr. Smit reports grants from FHI360, during the conduct of the study; No other authors report any conflicts of interest.

## Appendix Supplementary materials

Image, application 1

Image, application 1
