# Supplementary material for: Weight change among women using intramuscular depot medroxyprogesterone acetate, a copper intrauterine device, or a levonorgestrel implant for contraception: Findings from a randomised, multicentre, open-label trial
Source: eClinicalMedicine. 2021 Apr 6;34:100800. doi: 10.1016/j.eclinm.2021.100800 (PMC8056402; doi:10.1016/j.eclinm.2021.100800)
Supplement: Supplementary file 1 [file mmc1.docx]

**Supplementary Table 1: Shift table depicting baseline and final BMI category**
