# Supplementary material for: Weight change among women using intramuscular depot medroxyprogesterone acetate, a copper intrauterine device, or a levonorgestrel implant for contraception: Findings from a randomised, multicentre, open-label trial
Source: eClinicalMedicine. 2021 Apr 6;34:100800. doi: 10.1016/j.eclinm.2021.100800 (PMC8056402; doi:10.1016/j.eclinm.2021.100800)
Supplement: Supplementary file 2 [file mmc2.docx]

Supplementary Tables and Figures

**Supplementary Table 1: Shift table depicting baseline and final BMI category**

| **Method** | **BMI categories** | **Underweight** | **Normal** | **Pre-obesity** | **Obesity** | **Severe obesity** | **Morbid obesity** |
| --- | --- | --- | --- | --- | --- | --- | --- |
| DMPA-IM | **Underweight** | 57 | 52 | 0 | 0 | 0 | 0 |
|  | **Normal** | 32 | 715 | 256 | 16 | 0 | 0 |
|  | **Pre-obesity** | 0 | 52 | 331 | 187 | 11 | 0 |
|  | **Obesity** | 0 | 5 | 28 | 192 | 92 | 7 |
|  | **Severe obesity** | 0 | 0 | 0 | 14 | 94 | 61 |
|  | **Morbid obesity** | 0 | 0 | 0 | 0 | 10 | 81 |
| LNG Implant | **Underweight** | 51 | 42 | 0 | 0 | 0 | 0 |
|  | **Normal** | 38 | 761 | 185 | 7 | 0 | 0 |
|  | **Pre-obesity** | 0 | 75 | 385 | 154 | 13 | 0 |
|  | **Obesity** | 0 | 0 | 54 | 245 | 95 | 8 |
|  | **Severe obesity** | 0 | 0 | 0 | 22 | 105 | 42 |
|  | **Morbid obesity** | 0 | 0 | 0 | 0 | 12 | 78 |
| Copper IUD | **Underweight** | 67 | 35 | 0 | 0 | 0 | 0 |
|  | **Normal** | 36 | 811 | 171 | 4 | 0 | 0 |
|  | **Pre-obesity** | 0 | 88 | 404 | 117 | 7 | 0 |
|  | **Obesity** | 0 | 0 | 55 | 215 | 61 | 3 |
|  | **Severe obesity** | 0 | 0 | 0 | 31 | 103 | 40 |
|  | **Morbid obesity** | 0 | 0 | 0 | 0 | 7 | 94 |
